# Analysis of a Chronic Lateral Ankle Instability Model in the Rat: Conclusions and Suggestions for Future Research

**DOI:** 10.3390/life14070829

**Published:** 2024-06-28

**Authors:** Ibrahim Saliba, Manon Bachy-Razzouk, Morad Bensidhoum, Thierry Hoc, Esther Potier, Raphaël Vialle, Alexandre Hardy

**Affiliations:** 1 Orthopedics Department, Cochin Hospital, 75014 Paris, France; 2 Orthopedics Department, Armand Trousseau Hospital, 75012 Paris, France; manon.bachy@aphp.fr (M.B.-R.); raphael.vialle@aphp.fr (R.V.); 3 CNRS, INSERM, ENVA, B3OA, University of Paris Cite, 75010 Paris, France; morad.bensidhoum@u-paris.fr (M.B.); thierry.hoc@cnrs.fr (T.H.); esther.potier@cnrs.fr (E.P.); 4 Mechanical Department, Ecole Centrale—Lyon, 69134 Ecully, France; 5 Clinique Du Sport, 75005 Paris, France; alexandre.hardy@me.com

**Keywords:** chronic lateral ankle instability, surgically-induced osteoarthritis, rat model, lateral ankle ligaments

## Abstract

The purpose of this study was to evaluate potential osteoarthritic alterations within the ankle using a surgically-induced chronic lateral ankle instability (CLAI) model. Twelve rats were assigned randomly to either the control (*n* = 4) or CLAI group (*n* = 8). Surgery was performed on the right ankle. Osteoarthritis was assessed through in-vivo micro-CT at 8 weeks and a clinical analysis. Macroscopic analysis, high-resolution ex-vivo micro-CT and histological examination were conducted after euthanasia at 12 weeks. Three subgroups (SG) were analyzed. SG1 comprised the operated ankles of the CLAI group (*n* = 8). SG2 consisted of the non-operated ankles of the CLAI group (*n* = 8). SG3 included both sides of the control group (*n* = 8). In-vivo micro-CT revealed no significant differences among the three subgroups when analyzed together (*p* = 0.42), and when comparing SG1 with SG2 (*p* = 0.23) and SG3 (*p* = 0.43) individually. No noticeable clinical differences were observed. After euthanasia, macroscopic analysis employing OARSI score, did not demonstrate significant differences, except between the medial tibia of SG1 and SG3 (*p* = 0.03), and in the total score comparison between these two subgroups (*p* = 0.015). Ex-vivo micro-CT did not reveal any differences between the three subgroups regarding bony irregularities and BV/TV measurements (SG1 vs. SG2 vs. SG3: *p* = 0.72; SG1 vs. SG2: *p* = 0.80; SG1 vs. SG3: *p* = 0.72). Finally, there was no difference between the three subgroups regarding OARSI histologic score (*p* = 0.27). These findings indicate that the current model failed to induce significant osteoarthritis. However, they lay the groundwork for improving the model’s effectiveness and expanding its use in CLAI research, aiming to enhance understanding of this pathology and reduce unnecessary animal sacrifice.

## 1. Introduction

Ankle sprains are prevalent injuries observed among athletes and the general population [1,2,3,4]. They constitute between 10% and 30% of all sports-related injuries [5]. Inversion-type ankle sprains, accounting for 80% to 90% of cases, represent the most common mechanism of injury [3,5]. While the majority of lateral ankle ligamentous injuries demonstrate favorable outcomes with conservative treatment, a subset of patients, ranging from 10% to 30%, may develop a persistent condition known as Chronic Lateral Ankle Instability (CLAI) [1,6]. This condition is identifiable by persistent symptoms, such as pain, a sense of instability, and a feeling of giving way. Furthermore, it can lead to both short-term and long-term functional impairments, posing a potential risk to other anatomical structures that may necessitate surgical intervention, including osteochondral injuries and peroneal tendon pathologies [5,7].

Various surgical procedures are available to address CLAI, ranging from ligament repairs to reconstructions utilizing autograft or allograft tissue [2,5,8,9,10]. These surgical interventions aim to restore stability and function to the affected ankle joint. Currently, there exists conflicting evidence concerning the optimal timing for surgical intervention in patients with CLAI, as there is a wide range of variable delay in treatment following the initial injury [5,11]. For instance, some authors [12] advocate for immediate surgical intervention for patients with grade III ankle ligament injuries [13], which involve a complete tear of the lateral ankle ligament complex, while others recommend initial medical management and only advise surgical treatment if a well-conducted six-month course of medical management fails [14]. On the other hand, reports in the literature include experimental studies that demonstrate surgically-induced models of CLAI in mice [15] that, subsequently lead to the development of osteoarthritis after 8 weeks. Additionally, a surgical rat model to simulate acute ankle sprains was described, but it was not aimed at exploring the effects of CLAI [16].

The objective of the current study was to evaluate the impact on the ankle joint of a surgically-induced CLAI rat model. If this model proves effective in inducing osteoarthritis, it may enable the evaluation of interventions related to ankle instability and osteoarthritis, thereby enhancing the understanding of the associated pathology and potentially participating in advancing its treatment and management.

## 2. Materials and Methods

Twelve adult male Wistar rats (470–520 g, Janvier-Labs, Le Genest-Saint-Isle, France) were employed as the animal model for this IRB-approved experimental study (APAFIS #38860-2022091515149680 v7). The rats were housed in subgroups of three within plastic cages equipped with soft bedding material. The animal facility maintained a reversed 12-h light/dark cycle. The rats were provided with a standard diet and had unrestricted access to water. The environmental conditions in which the rats were housed included an ambient temperature of 22 °C and a relative humidity ranging from 55% to 60%. These conditions were maintained within a pathogen-free environment in compliance with established protocols.

All animals were assigned randomly to one of two groups: the CLAI group (*n* = 8 rats), in which the right side anterior talofibular ligament (ATFL) and calcaneo-fibular ligament (CFL) were surgically resected, or the control group (*n* = 4 rats). Each cage housed two rats from the CLAI subgroup and one rat from the control subgroup. To distinguish between the animals, their tails were marked using a marker pen. Specifically, Rat 1 (R1) in each cage had a single mark on its tail, R2 had two marks, while R3 (control) had no marks. The ankles of these animals were categorized into three distinct subgroups. Subgroup 1 (SG1) consisted of the surgically operated right ankles of the CLAI group, comprising a total of 8 ankles. Subgroup 2 (SG2) included the non-operated left ankles of the CLAI group animals, also comprising 8 ankles. Subgroup 3 (SG3) encompassed both ankles (right and left) of the rats in the control group, totaling 8 ankles.

### 2.1. Surgery

#### 2.1.1. Preparation

Two hours prior to the surgical procedure, the rats were anesthetized using 4% isoflurane. Tail marking was performed for individual identification, and the rats were weighed to record their baseline weights. The right ankle was shaved to ensure clear visibility. Subsequently, a subcutaneous injection of buprenorphine (0.16 mg/kg) and an intramuscular injection of meloxicam (1 mg/kg) were administered to provide analgesia. Following this, the rats were returned to their original cages to allow for a suitable preoperative period.

#### 2.1.2. Surgical Procedure

Following induction of anesthesia with isoflurane, the rat was positioned in supine position with a slight tilt on the left side to optimize exposure of the right side. Aseptic measures were carried out using a solution of povidone iodine (Betadine).

Palpation was performed to locate the lateral malleolus and the base of the 5th metatarsus. A size 11 blade was used to make an incision in the skin, while simultaneously raising the skin using claw forceps. The incision extended approximately 2 cm from the lateral malleolus to the base of the 4th metatarsus.

Special care was taken during the incision to avoid damage to deep structures. Adhesions were carefully sectioned along with the subcutaneous tissue. The fibular tendons were identified, and dissection was conducted posteriorly to expose them.

**Figure 1:**
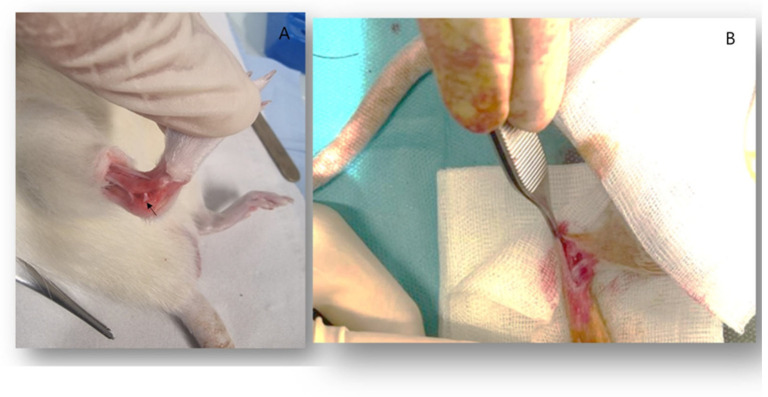
(**A**) showing the CFL in place (arrow). (**B**) showing the ankle joint during surgery after resection of CFL.

Using a blade and Halstead forceps, a gentle dissection was carried out in the talo-calcaneal space, while dorsiflexing and inverting the ankle to clearly visualize the CFL (Figure 1A). The CFL was sectioned using a size 11 blade (Figure 1B).

**Figure 2:**
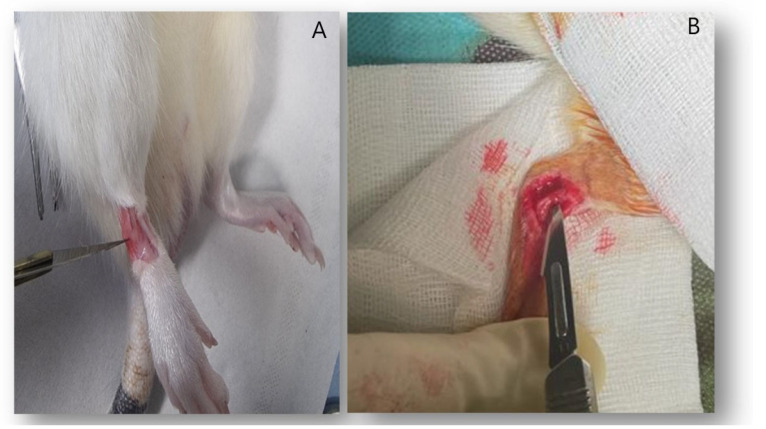
(**A**) showing the ATFL in place between the extensors anteromedially and the peroneal tendons laterally. (**B**) showing the resection of ATFL during surgery.

Next, the extensors located anteriorly and the lateral peroneal muscles were identified (Figure 2A). Plantarflexion and inversion of the ankle were performed, and the blade was gently inserted inside the lateral malleolus. The blade was then rotated clockwise to cut the ATFL while being cautious not to damage the fibular tendons (Figure 2B).

After irrigation with normal saline, the skin was closed using either separate stitches with non-absorbable suture material. No dressing was applied postoperatively.

#### 2.1.3. Postoperative Care

Postoperative care for the rats followed a structured protocol. The protocol consisted of the following steps:

Day 0 (D0): Upon discontinuation of isoflurane anesthesia, the rats were monitored for awakening. In the evening, a second subcutaneous injection of Buprenorphine was administered.

D1 to D3: The rats received buprenorphine subcutaneously twice daily, and meloxicam was administered intramuscularly once daily, with observation for signs of pain, mobility, and well-being.

From D7 to D60, the rats were monitored twice a week, with regular redrawing of the tail mark to prevent fading and ensure continuous identification. Monitoring included assessing parameters such as weight, general well-being, and the ability to bear weight and stand.

### 2.2. In-Vivo Micro CT

**Figure 3:**
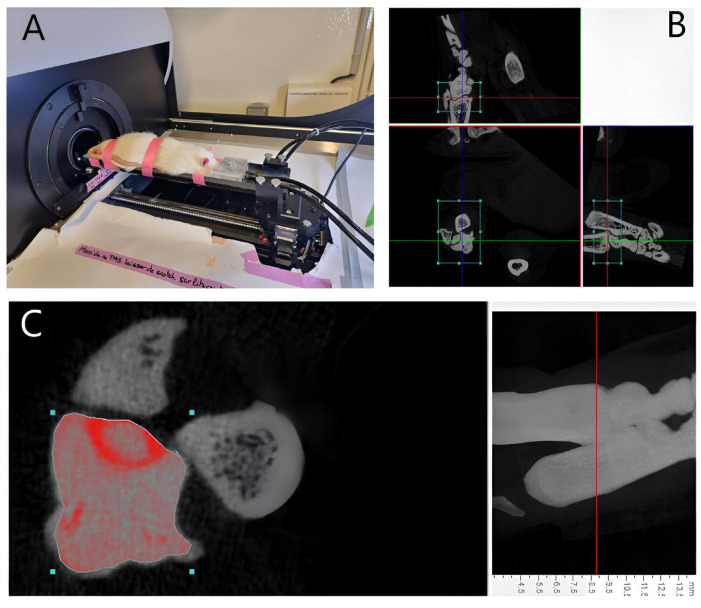
(**A**) Showing a rat under anesthesia undergoing in-vivo micro CT at 8 weeks. (**B**) Three-dimensional reconstruction of the region of interest representing the tibiotalar joint (rectangle) using DataViewer software version 1.5.4.0 64-bit; (**C**) Measurement of the tibiotalar joint space volume using CtAn software version 1.17.7.2+ (Scale bar = 1 mm).

At 8 weeks postoperatively, a Micro CT examination (SkyScan1173, Bruker-CT: source voltage (kV) = 65; source current (uA) = 313; number of rows = 668; number of columns = 1000; image pixel size (um) = 35.18) was conducted to assess the presence of radiological indicators associated with tibio-talar osteoarthritis of the ankle. The rats were positioned in a prone position and placed under general anesthesia using 4% isoflurane at a flow rate of 2 to 3 L/min (Figure 3A). To facilitate accurate interpretation of the images and avoid confusion between sides, a hydroxyapatite marker was affixed to the right foot, which had undergone the surgical procedure. This marker served as a spatial reference point during image analysis. The extent of articular cartilage damage was assessed by measuring the volume of the tibio-talar joint in all ankles belonging to SG1, SG2, and SG3. This was achieved by selecting the tibio-talar joint as the region of interest, quantifying the total tissue volume within this region, and extracting the bone volume from the tissue volume. The remaining volume represented the joint volume between the distal tibia and the talus (Figure 3B,C).

### 2.3. Clinical Observation

In addition to the routine weekly observations, close qualitative examination of the rats was conducted by two reliable observers prior to sacrifice. The purpose was to meticulously evaluate any discernible clinical variations among the animals, particularly in terms of behavior, weight-bearing capacity, and ability to stand up. Any presence of limping or uneven weight distribution between legs was carefully documented. Furthermore, food placement at the top of the cage prompted the rats to stand upright on their hind limbs to access it, allowing observation of any abnormal weight-bearing patterns or disparities between the operated and non-operated legs, which were also duly recorded.

### 2.4. Ex-Vivo Micro CT

After sacrifice, radiologic changes in the ankle OA were evaluated using a high-resolution micro-CT (SkyScan1173; Bruker-CT). After fixing the specimen to a jig for micro-CT measurement using parafilm, 800 image images were acquired using a tube voltage of 65 kV, a current of 313 µA, and a 1.0 aluminum filter.

The cross-section was reconstructed using NRecon software version 1.7.4.2 (Bruker, Kontich, Belgium). The obtained cross-sectional images were aligned for each cross-section using DataViewer version 1.5.4.0 64-bit (Bruker, Kontich, Belgium), and the parameter values were calculated using CtAn software version 1.17.7.2+ (Bruker, Kontich, Belgium). The high-resolution images acquired through ex-vivo micro CT examination were intended for the assessment of bone volume fraction (BV/TV; where BV denotes Bone Volume and TV denotes Tissue Volume). This parameter is recognized to exhibit an increase in osteoarthritis models [15]. Additionally, the images were utilized for the identification of osteophytes or gross bony irregularities, which are commonly observed manifestations in osteoarthritis.

### 2.5. Macroscopic Analysis

**Figure 4:**
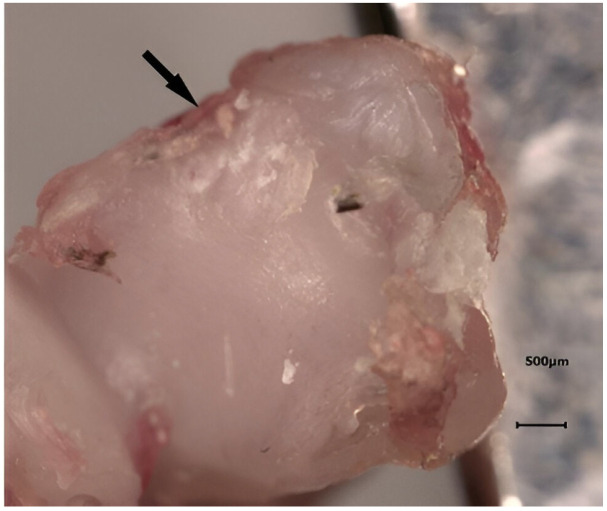
Macroscopic analysis of a distal tibia specimen from an operated rat: the anteromedial cartilage damage can be clearly seen (arrow).

Using BZ II analyzer software in BZ-8100 (KEYENCE, Osaka, Japan), macroscopic assessment of distal tibia and talus specimens was performed (Figure 4), and the OARSI macroscopic scoring [17,18] system was utilized. An independent orthopedic surgeon, who was blinded to the experimental subgroups, conducted evaluations of the distal tibia and talus specimens to detect any manifestations of articular or cartilage damage. Each distal tibia or talus specimen underwent grading for both the medial and lateral articular surfaces, employing a scale ranging from 0 (indicating the absence of gross fibrillation or fissuring) to 4 (representing extensive full-thickness erosions extending to the level of subchondral bone).

### 2.6. Histological Analysis

The talus of each ankle joint was fixed with 10% neutral buffered formalin for 24 h, decalcified for one week in OSTEOSOFT^®^ at 37 °C on a shaker for 5 days, and then embedded in paraffin. The specimens were then stained with safranin O, fast green, and Toluidine blue according to the standard protocol. The OARSI histologic scoring system was used in histopathological assessment of the specimens [19]. For each specimen, the microscopic score was ascertained through the multiplication of the assigned grade and stage values. The grade represented the depth of lesions, ranging from superficial cartilage to pathological alterations in the subchondral bone. Concurrently, the stage delineated the extent of osteoarthritic lesions with regard to surface involvement.

### 2.7. Outcome Measures

In summary, the study outcomes included osteoarthritis-related measures, such as joint volume (evaluated via in vivo micro-CT at 8 weeks), OARSI macroscopic and microscopic scoring (at 12 weeks), and osteophyte and bone irregularity assessment (at 12 weeks using macroscopic evaluation and ex vivo micro-CT), as well as bone volume fraction (BV/TV) evaluated by ex vivo micro-CT at 12 weeks. Clinical outcomes were observed through behavior, weight-bearing capacity, and the rats’ ability to stand up post-surgery.

### 2.8. Statistical Analysis

Statistical analyses were conducted using version 3.5.0 of R software available at https://www.R-project.org accessed on 5 September 2023. Quantitative variables were described using means and standard deviations. For comparisons between two subgroups of quantitative variables, the Mann-Whitney test was employed. When more than two subgroups were compared, the Kruskal-Wallis test was utilized. A significance level of *p* < 0.05 was considered statistically significant.

## 3. Results

At the final follow up (before sacrifice), there was no mortality nor other surgical complications among the animals.

### 3.1. In-Vivo Micro CT

The mean joint volume was found to be 1.76 mm^3^ in SG1, 2.25 mm^3^ in SG2, and 2.16 mm^3^ in SG3. Statistical analysis revealed no significant difference between SG1 and SG2 (*p* = 0.23), nor between SG1 and SG3 (*p* = 0.43). Furthermore, no significant difference was observed when comparing SG1, SG2, and SG3 collectively (*p* = 0.42).

### 3.2. Clinical Observation

The two observers independently reported that there were no notable differences observed between the rats that underwent the surgical procedure and those that did not.

### 3.3. Macroscopic Analysis

**Figure 5:**
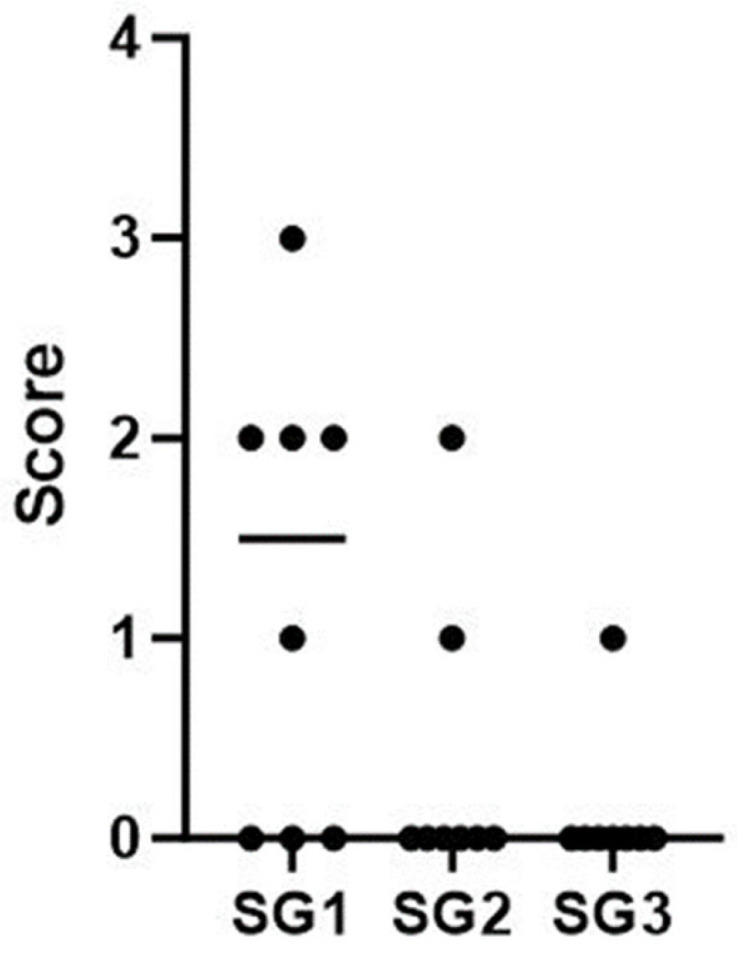
Distribution of macroscopic score values for the medial tibia in the three subgroups SG1, SG2, and SG3.

**Figure 6:**
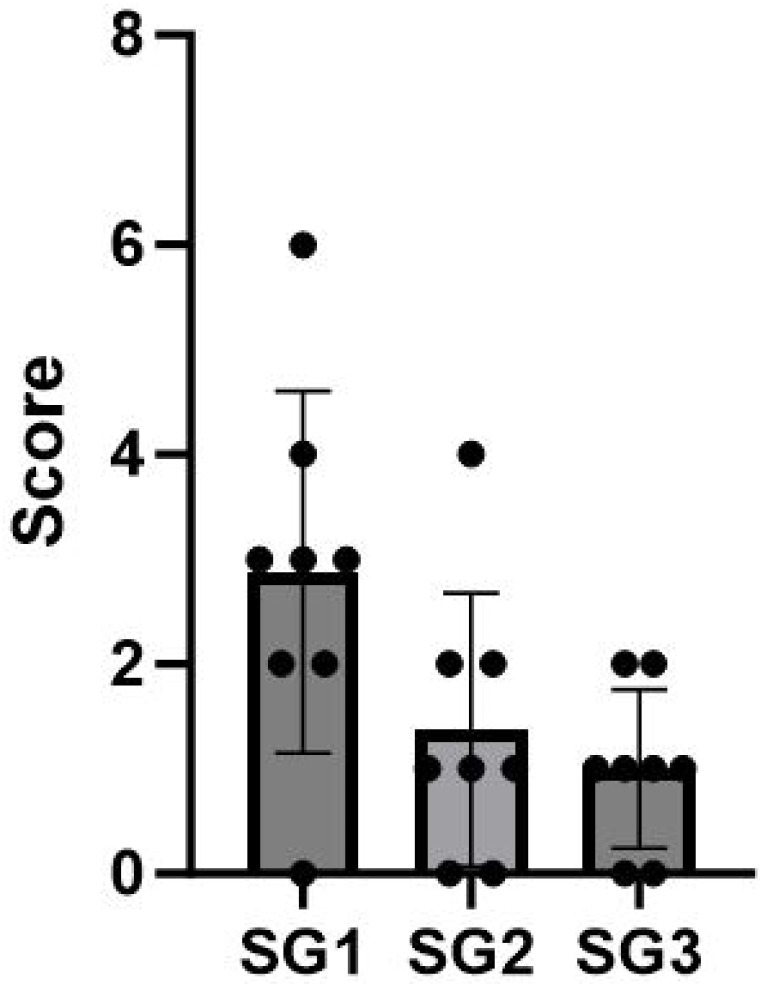
Distribution of total macroscopic score values for each ankle in the three subgroups SG1, SG2, and SG3.

The results indicated no statistically significant differences among the subgroups, except for the medial tibias of the SG1 and SG3 subgroups, where the mean scores were SG1 = 1.25 and SG3 = 0.125, respectively (*p* = 0.03) (Figure 5 and Table 1). Furthermore, a statistically significant difference was observed in the total score comparison between SG1 and SG3, with mean scores of SG1 = 2.9 and SG3 = 1 (*p* = 0.015) (Figure 6 and Table 2). It is important to emphasize that the assessor did not detect any osteophytes or gross articular damage during his evaluation.

### 3.4. Ex-Vivo Micro CT

The mean BV/TV was measured to be 9.3 in SG1, 9.8 in SG2, and 9.1 in SG3.

**Figure 7:**
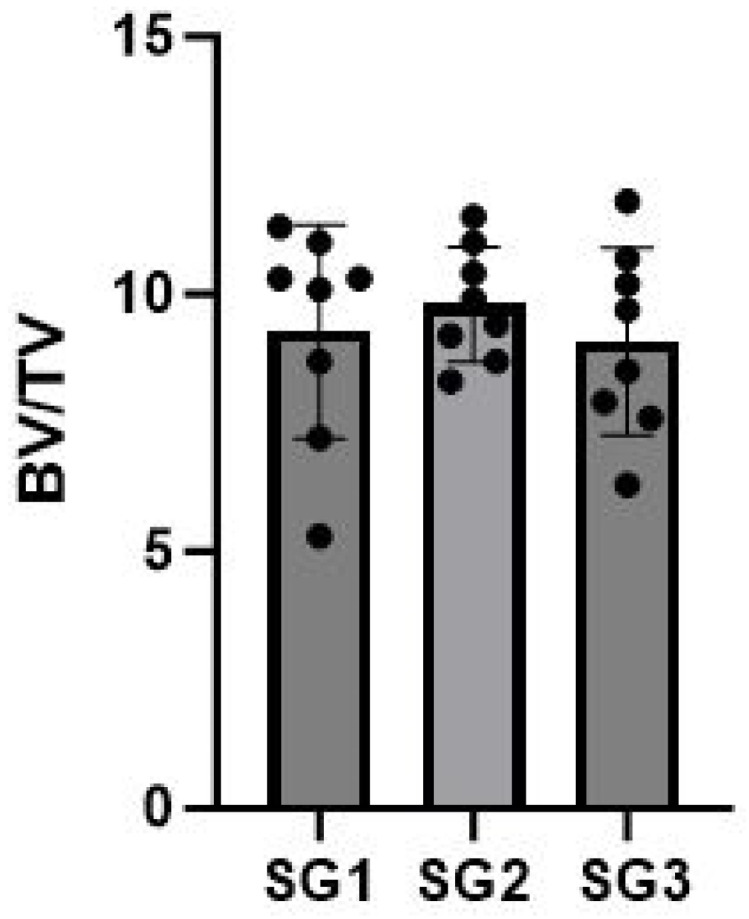
Distribution of Bone Volume to Tissue Volume (BV/TV) values in the three subgroups SG1, SG2, and SG3.

Statistical analysis revealed no significant differences in BV/TV when comparing SG1 to SG2 (*p* = 0.80) and SG1 to SG3 (*p* = 0.72), respectively. Additionally, when comparing all three subgroups (SG1, SG2, and SG3) collectively, there were no significant differences observed (*p* = 0.72) (Figure 7 and Table 3).

**Figure 8:**
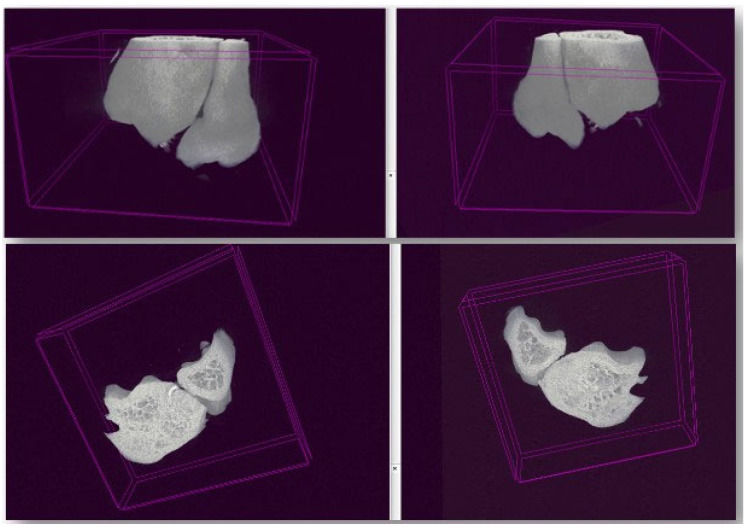
Ex-vivo micro-CT of samples from operated rats with 3D reconstruction using CT Vox Version 3.3.0 (64-bit). No bony irregularities or osteophytes were identified.

Regarding the presence of gross bony irregularities or osteophytes, the examination of specimens from all three subgroups yielded consistent results, indicating an absence of these characteristic signs associated with osteoarthritis (Figure 8).

### 3.5. Histopathological Assessment

**Figure 9:**
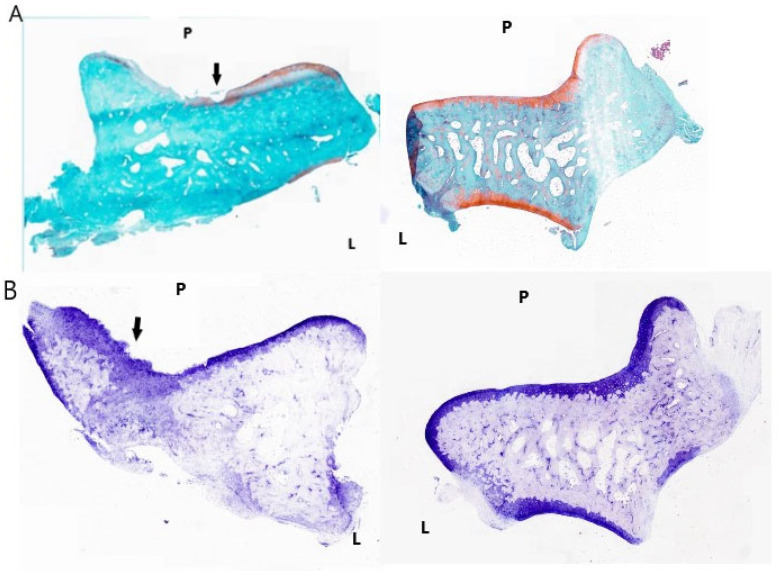
(**A**) Left photo: operated side talus of R1C1 rat of the CLAI group. This specimen was stained with safranin-O and fast green. The black arrow indicates the erosion of the cartilage exposing the subchondral bone. Right photo: non-operated side talus of the same animal, stained with safranin-O and fast green, showing normal aspect without cartilage or bone defects. (**B**) left photo: operated side talus of R2C3 rat of the CLAI group. This specimen was stained with Toluidine blue. The black arrow indicates the severe erosion of the cartilage layer with denudation, exposing the subchondral bone. Right photo: non-operated side talus of R2C3 rat, stained with Toluidine blue. There are no cartilage or subchondral bone defects. P: proximal; L: lateral.

The specimens were examined by a blinded pathologist and evaluated utilizing the OARSI microscopic grading system [19]. There were no significant differences regarding cartilage damage and subchondral bone pathology between the 3 subgroups (*p* = 0.27) (Figure 9 and Table 4).

## 4. Discussion

The present study effectively established a rat model of surgically induced post-traumatic CLAI without eliciting significant consistent osteoarthritic changes. A previously reported rat model related to surgically-induced ankle instability focused on ankle sprain to test the effects of morphine and indomethacin in this pathology [16]. Notably, that surgically-induced model of ankle instability was successfully realized in mice [15], leading to the development of osteoarthritis within 8 to 12 weeks. In those mice studies, high-resolution micro-CT evaluation was performed post-sacrifice to assess the specimens. In that research, an in-vivo micro-CT was conducted at 8 weeks to determine whether any signs of osteoarthritis were present, guiding the decision on whether to sacrifice the rats in the current study at 8 or 12 weeks due to the lack of prior literature data on the rat CLAI model.

The in-vivo micro-CT findings at 8 weeks did not reveal any differences between subgroups, prompting the planned sacrifice of animals at 12 weeks. Clinical observations before euthanasia were consistent with the in-vivo micro-CT results. While the weight bearing ratio (WBR) has been proposed as a clinical tool for assessing ankle instability models [20,21], it was not used in this study. Instead, thorough observations by two independent observers concurred that there were no clinical differences between subgroups. WBR devices were not utilized because they measure weight-bearing force during locomotion and gait analysis [22,23]. Instead, abnormal weight distribution was detected qualitatively by reliable observers. Ratios of weight-bearing force distribution were deemed unnecessary, as the objective of this study was not to evaluate or compare painkillers but to identify pronounced ankle osteoarthritis, which is clinically evident without quantifying force distribution. Moreover, current WBR measurement assays do not assess weight distribution in rodents while standing at rest in a normal (pronograde) posture [22,24,25,26], an important aspect for this study due to the impact of asymmetry on the hindlimbs.

The macroscopic analysis of the specimens after sacrifice demonstrated no significant differences between subgroups, except for the medial tibia of SG1 and SG3 and the total scores for SG1 and SG3, respectively. A possible explanation for this finding is that the operated rats tended to place more weight-bearing force on the non-operated ankles (SG2) than on the operated ankles (SG1). This compensatory behavior may have led to alterations in the non-operated ankles (SG2), resulting in scores for this subgroup that were closer to those of SG1. This may explain the lack of significant difference between SG1 and SG2. In contrast, a significant difference was observed between SG1 and SG3, as the latter subgroup exhibited equivalent and symmetrical weight-bearing distribution, with no alterations in the ankle joints, leading to much lower scores in SG3 compared with SG1.

The abovementioned findings could be attributed to the fact that the ankle instability model led to mild cartilage damage, which was observed macroscopically but did not progress to advanced osteoarthritis with severe cartilage and subchondral bone damage. Moreover, high-resolution ex-vivo micro-CT analysis did not reveal any differences in BV/TV between subgroups.

Histopathological evaluation of specimens showed the surgically induced ankle instability model’s effect on mild cartilage damage without affecting the subchondral bone. These osteoarthritic changes were not constantly observed in the operated rats explaining the absence of any significant difference between subgroups.

To the best of our knowledge, this is the first experimental study to have assessed this specific model in rats. However, the current form of this model may not be suitable for investigating management and treatment strategies for ankle instability that are aimed at reducing the risk of developing osteoarthritis. Initially, our plan was to validate this model to determine whether it led to significant osteoarthritis. If this condition had been met, we intended to compare the efficacy of early versus late ligament repair and to evaluate the potential benefits of early repair in preventing osteoarthritis. However, we did not proceed to ligament repair because this model was not validated. To potentially enhance the model’s efficacy, we propose the additional resection of the posterior talofibular ligament (PTFL) alongside the ATFL and CFL. Our observations align with a report in the literature indicating that the ATFL may not significantly contribute to ankle stability during movement in rats [16]. This latter report’s results suggested that PTFL and CFL may be the two main stabilizing ligaments of the lateral ankle in rats [16]. Moreover, rodents exhibit significant regenerative capabilities, allowing injured ligaments to heal rapidly [27]. Therefore, we propose the complete removal of the ligaments, without leaving remnants, in conjunction with the excision of the joint capsule, to reliably induce ankle instability.

Another suggestion to improve the model’s efficacy is to increase the animals’ locomotor activity, for instance, by utilizing running wheels, and conducting an in-vivo micro-CT evaluation at 12 weeks. If no significant changes are observed at this time point, prolonging the postoperative follow-up to 24 weeks could be considered.

The future validation of this model in rats is crucial since certain interventions related to ankle instability and osteoarthritis may be challenging to evaluate in mice. This rat model could serve as an alternative when mice models are impractical for such interventions. One limitation of this study is that the assessments involving in-vivo micro-CT, ex-vivo micro-CT, and macroscopic scoring of specimens were conducted only once. Furthermore, additional parameters beyond BV/TV could have been analyzed using ex-vivo micro-CT [28]. These parameters include those related to bony microarchitecture, such as cortical bone data (bone cross-sectional area, total mean bone cross-sectional area) and trabecular bone data (trabecular thickness, trabecular number, and trabecular separation) [28]. The objective of this study was to determine whether this model can induce severe osteoarthritis of the ankle joint, as evidenced by a significant reduction in joint space volume and the presence of osteophytes and gross bone anomalies. However, it is also important to analyze modifications in microarchitecture. This aspect should be included in the revised model for future studies. Moreover, the macroscopic scoring was performed by a single individual, potentially raising concerns about measurement reliability. Nevertheless, the consistency of these assessments with the results of the histological study strengthens the validity of the research findings.

## 5. Conclusions

In conclusion, this study established a surgically-induced CLAI model in rats by resecting the ATFL and CFL, resulting in mild cartilage damage without affecting the subchondral bone. To enhance the model’s potential, inclusion of PTFL resection and increased locomotor activity is recommended. This rat model, effective in inducing osteoarthritis, allows the assessment of interventions related to ankle instability and osteoarthritis when mouse models are unsuitable. These findings lay the groundwork for further refinement, improving the model’s efficacy, broadening its applications, and contributing to a comprehensive understanding of CLAI and its management while reducing unnecessary animal sacrifice. The clinical relevance lies in providing a robust platform for testing therapeutic strategies for ankle instability and osteoarthritis.

## Figures and Tables

**Table 1:** This table depicts the macroscopic score values for the medial tibia of animals in the three subgroups. Regarding SG3, the value above corresponds to the right side, while the value below corresponds to the left side.

SG	Rat	Macroscopicscore (Medial Tibia)	Mean [Min–Max]
SG1 (Right side)	R1C1	3	
	R2C1	0	
	R1C2	0	
	R2C2	2	
	R1C3	1	
	R2C3	0	
	R1C4	2	
	R2C4	2	1.3 [0–3]
SG2 (Left side)	R1C1	0	
	R2C1	2	
	R1C2	0	
	R2C2	0	
	R1C3	0	
	R2C3	0	
	R1C4	0	
	R2C4	1	0.4 [0–2]
SG3 (Control group)	R3C1	1	
		0	
	R3C2	0	
		0	
	R3C3	0	
		0	
	R3C4	0	
		0	0.1 [0, 1]

**Table 2:** This table illustrates the total macroscopic score values for the ankles in the three subgroups. In the case of SG3, the upper value corresponds to the right side, while the lower value corresponds to the left side.

SG	Rat	Macroscopic Score (Total Score)	Mean [Min–Max]
SG1 (Right side)	R1C1	6	
	R2C1	2	
	R1C2	0	
	R2C2	4	
	R1C3	3	
	R2C3	2	
	R1C4	3	
	R2C4	3	2.9 [0–6]
SG2 (Left side)	R1C1	2	
	R2C1	4	
	R1C2	1	
	R2C2	1	
	R1C3	1	
	R2C3	0	
	R1C4	0	
	R2C4	2	1.4 [0–4]
SG3 (Control rats)	R3C1	2	
		1	
	R3C2	1	
		0	
	R3C3	2	
		1	
	R3C4	1	
		0	1 [0–2]

**Table 3:** The Bone Volume to Tissue Volume (BV/TV) values of the samples from the three subgroups. For SG3, the upper value corresponds to the right side, while the lower value corresponds to the left side.

SG	Rat	BV/TV	Mean [Min–Max]
SG1 (Right talus)	R1C1	11.3	
	R2C1	10.3	
	R1C2	10.3	
	R2C2	11.0	
	R1C3	8.7	
	R2C3	10.1	
	R1C4	5.3	
	R2C4	7.2	9.3 [5.3–11.3]
SG2 (Left talus)	R1C1	11.5	
	R2C1	9.4	
	R1C2	11.0	
	R2C2	9.2	
	R1C3	8.3	
	R2C3	9.9	
	R1C4	8.7	
	R2C4	10.4	9.8 [8.3–11.5]
SG3 (Talus on both sides)	R3C1	6.3	
		9.7	
	R3C2	7.6	
		10.2	
	R3C3	10.7	
		8.5	
	R3C4	11.8	
		7.9	9 [6.3–11.8]

**Table 4:** The microscopic score values of the talus bone samples from the three subgroups. For SG3, the upper value corresponds to the right side, while the lower value corresponds to the left side.

SG	Rat	Microscopic Score	Mean [Min–Max]
SG1 (Right talus)	R1C1	5	
	R2C1	0	
	R1C2	8	
	R2C2	3	
	R1C3	0	
	R2C3	0	
	R1C4	0	
	R2C4	8	3.0 [0–8]
SG2 (Left talus)	R1C1	0	
	R2C1	0	
	R1C2	0	
	R2C2	0	
	R1C3	3	
	R2C3	0	
	R1C4	0	
	R2C4	0	0.4 [0–3]
SG3 (Talus on both sides)	R3C1	0	
		0	
	R3C2	0	
		0	
	R3C3	0	
		0	
	R3C4	0	
		3	0.4 [0–3]

## Data Availability

Data are contained within the article.
